# Active pH Modulation by Proton Channel‐Peptide Nucleic Acid Complex for Effective siRNA Endosomal Escape

**DOI:** 10.1002/smll.74802

**Published:** 2026-07-23

**Authors:** Seongmin Ga, Nam Hyeong Kim, Si Yeon Ryu, Kyeonghyun Kim, Eun Sung Kang, Hayeon Bae, Seungmin Ryu, Suhyeon Kim, Yeontae Jang, Bok‐Soo Lee, Jiyoung Nam, Minah Suh, Jaecheol Lee, Seung‐gu Kang, Jaekyung Hyun, Huong T. Kratochvil, Yong Ho Kim

**Affiliations:** ^1^ SKKU Advanced Institute of Nanotechnology (SAINT) Sungkyunkwan University (SKKU) Suwon Gyeonggi‐do Republic of Korea; ^2^ Department of Nano Science and Technology Sungkyunkwan University (SKKU) Suwon Gyeonggi‐do Republic of Korea; ^3^ Department of Pharmaceutical Chemistry University of California San Francisco (UCSF) San Francisco California United States; ^4^ Department of Biomedical Engineering Sungkyunkwan University (SKKU) Suwon Republic of Korea; ^5^ Center for Neuroscience Imaging Research (CNIR) Institute for Basic Science (IBS) Suwon Republic of Korea; ^6^ School of Pharmacy Sungkyunkwan University Suwon Republic of Korea; ^7^ IMNEWRUN INC. Suwon Republic of Korea; ^8^ Department of Nano Engineering Sungkyunkwan University Suwon Republic of Korea; ^9^ Department of MetaBioHealth Sungkyunkwan University Suwon Republic of Korea; ^10^ Department of Chemistry College of Arts and Sciences University of North Carolina at Chapel Hill Chapel Hill North Carolina United States

**Keywords:** endosomal escape, M2 proton channel, multiplexed gene silencing, peptide nucleic acid, siRNA drug

## Abstract

Small interfering RNA (siRNA) has shown great potential for treating various genetic diseases, but lysosomal degradation limits its bioavailability. Here, we present the design of a Channel‐PNA‐siRNA (CPR) complex that facilitates endosomal escape by actively inducing osmotic rupture of the endosomes. Our design utilizes the transmembrane domain of the M2 proton channel (M2TM) to facilitate proton influx into the liposome while serving as an anchor for siRNA; this surface tethering is mediated by a peptide nucleic acid (PNA) linker that enables stable, non‐covalent binding. Our results indicate that the CPR complex interrupts the endosomal pathway, effectively reducing the colocalization of delivered siRNA with lysosomes and enhancing gene regulation efficiency. Furthermore, we demonstrate dual‐target gene knockdown using CPR‐tethered liposomes by encapsulating additional siRNAs in the empty cavity of the liposome. These findings highlight the therapeutic potential of modulating the endosomal pH environment to prevent lysosomal degradation, suggesting that the CPR nanocarrier design could serve as a versatile platform for targeted RNA delivery.

## Graphical abstract

A bioinspired siRNA nanocarrier is engineered by integrating an influenza A M2 proton channel with siRNA through a peptide nucleic acid linker on liposome surfaces. Active proton transport amplifies osmotic stress within endosomes, triggering membrane rupture and cytosolic release. This channel‐driven pH modulation enables efficient and multiplexed gene silencing beyond conventional passive delivery strategies.

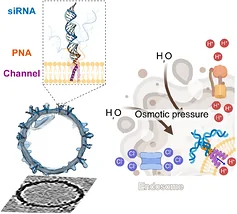

## 1 Introduction

RNA interference (RNAi), a fundamental cellular mechanism regulating gene activity, selectively suppresses specific genes by interfering with mRNA expression via small RNA molecules like small interfering RNAs (siRNAs) [1, 2, 3, 4]. siRNA drugs are promising therapeutic strategies for diverse diseases, including genetic conditions, viral infections, and various cancer types [5, 6, 7]. However, the clinical translation of RNAi has encountered persistent challenges over the past decade, primarily due to biological barriers hindering the sufficient intracytosolic transport of small RNA molecules. Given their substantial size and negative charge, siRNAs cannot directly penetrate the cell membrane [4, 8, 9]. Consequently, they rely on delivery systems such as endocytic liposomes, polymers, and nanoparticles [10], but their entrapment in endosomal compartments leads to accelerated degradation. Under the harsh acidic conditions during endosome‐lysosome trafficking, less than 2% of siRNAs escape from endosomes [11, 12, 13, 14]. Overcoming endosomal entrapment remains a critical bottleneck for the widespread application of RNA therapeutics in human disease treatment [15, 16, 17]. Recent studies have highlighted that only a small fraction of internalized siRNA successfully escapes the endo/lysosomal pathway, emphasizing the importance of engineering active endosomal escape strategies for efficient cytosolic delivery [18].

Active modulation of endosomal pH has emerged as a promising strategy to improve cytosolic siRNA delivery efficiency. Recent multifunctional nanocarrier systems have demonstrated that active intracellular trafficking modulation and enhanced lysosomal escape can significantly improve RNA delivery efficacy and intracellular bioavailability [19, 20, 21, 22]. Drawing inspiration from nature, various bioinspired modifications have been explored to enhance therapeutic efficiency in complex environments [23, 24]. Viruses, for example, employ pH‐mediated mechanisms to deliver their genetic material into the cytosol [25, 26]. In particular, the influenza A virus (IAV) utilizes the pH‐dependent M2 proton channel that unidirectionally conducts protons into the viral lumen [27, 28, 29, 30, 31, 32, 33]. This mechanism not only accelerates the endo‐lysosomal pathway but triggers structural changes in the hemagglutinin protein, facilitating fusion with the endosomal membrane [34, 35, 36]. Inspired by this natural mechanism, we propose incorporating the M2 proton channel into siRNA delivery carriers to induce the proton sponge effect, leading to the osmotic rupture of endosomes or lysosomes through the influx of chloride ions (Cl^−^) and water [37].

In this study, we designed a Channel‐PNA‐siRNA (CPR) complex to enhance the therapeutic efficacy of siRNA by actively facilitating proton transport from the endosome to the siRNA carrier. The complex was constructed by linking the transmembrane domain of the M2 proton channel (M2TM) to siRNA via a peptide nucleic acid (PNA) linker. These complexes were subsequently incorporated into liposomes, with siRNA displayed on the outer surface (CPR@LS). CPR@LS showed significantly enhanced endosomal escape compared to the control vehicle lacking proton‐conducting functionality. Furthermore, the encapsulation of additional siRNA payloads within liposomes containing the CPR complex enabled multiplexed gene silencing, targeting two distinct gene sequences simultaneously. Collectively, these findings underscore the therapeutic potential of this designed siRNA nanocarrier system in addressing a broad spectrum of genetic diseases.

## 2 Results and Discussion

### 2.1 Characterization of CPR Complex on Liposome

**FIGURE 1:**
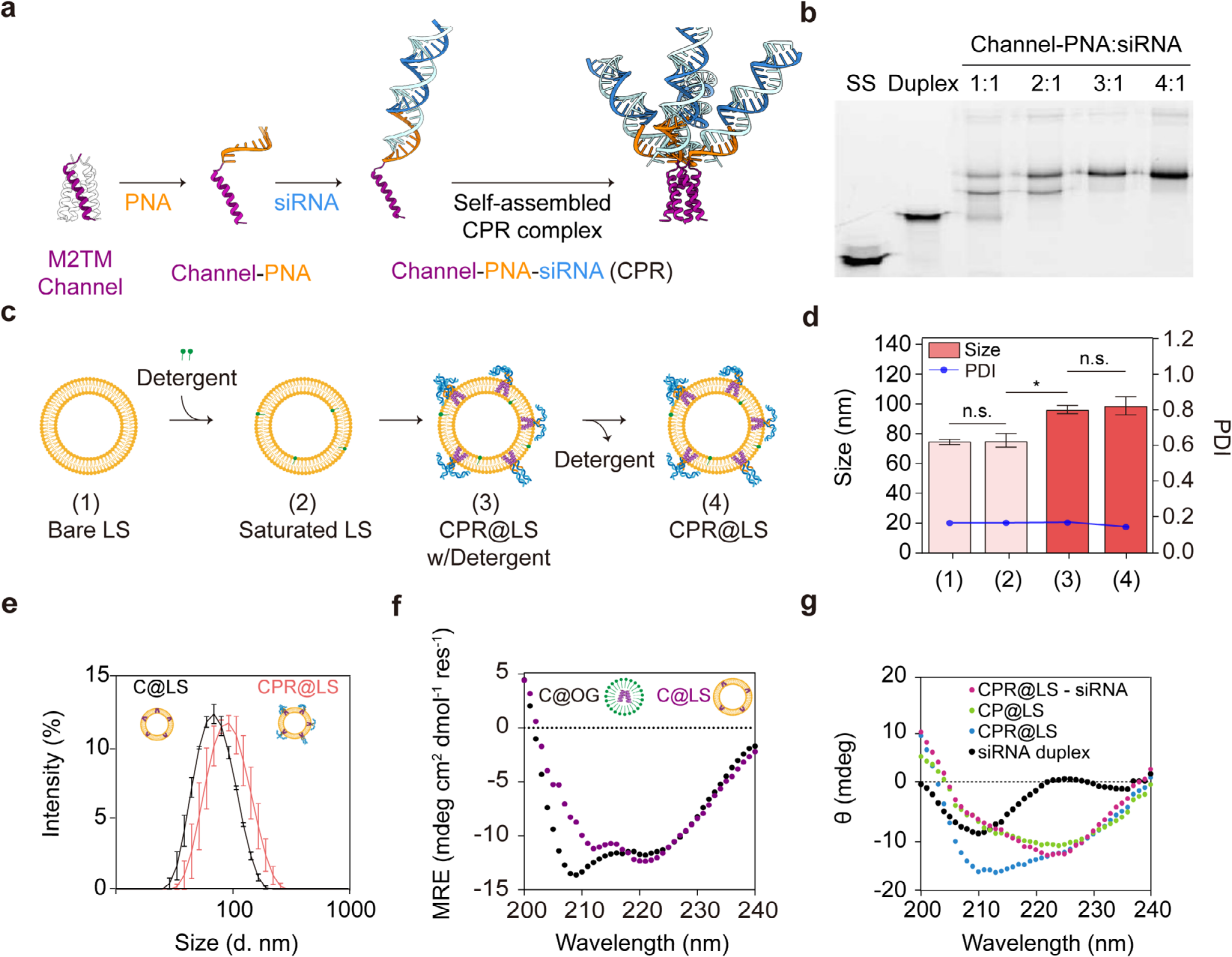
Characterization of CPR@LS inspired by Influenza A virus. a) Schematic illustration of Channel‐PNA‐siRNA (CPR) complex. b) Complexation of M2TM‐PNA and siRNA, observed on a 20% SDS gel, where SS indicates the sense strand. A 3:1 Channel‐PNA:siRNA molar ratio was used for the final CPR@LS formulation. c) Schematic illustration of the detergent‐mediated reconstitution process for the formation of CPR@LS, comprising four steps: 1) liposome preparation, 2) saturation of the liposome with detergent, 3) embedding CPR dissolved in detergent into the liposome, 4) removal of detergent using Bio‐bead. d) Hydrodynamic diameters (DH) and polydispersity index (PDI) values of liposomes at each step of the detergent‐mediated process. e) Hydrodynamic diameter (DH) comparison between C@LS and CPR@LS. f) comparison of helical spectra of M2 dissolved in detergent (C@OG) and M2 embedded in liposome (C@LS). g) CD spectra comparison of siRNA duplex, M2‐PNA, and CPR complex embedded in liposome (CP@LS and CPR@LS), along with the subtraction of the siRNA duplex spectrum from the CPR@LS spectrum (CPR@LS‐siRNA duplex). Statistical analysis was conducted using one‐way ANOVA. *P < 0.05, n.s.:no significant difference.

We selected the M2 transmembrane domain (M2TM, residues 22–46) as a minimal motif for stable proton channel formation in lipid bilayers [27, 38]. To facilitate non‐covalent siRNA loading, a peptide nucleic acid (PNA) was fused to the M2TM to allow sequence‐specific hybridization [39, 40]. Since PNA possesses a neutral amide‐like backbone rather than the negatively charged phosphodiester backbone of nucleic acids, PNA‐RNA hybrids generally exhibit stronger binding affinity and higher thermal stability than conventional nucleic acid duplexes [41, 42]. Taking advantage of these properties, we designed the PNA sequence to maintain stable hybridization under physiological conditions while preserving sufficient aqueous solubility. Specifically, the sequence was selected with guanine and purine contents below 3% and 50%, respectively, to minimize aggregation and ensure favorable solution behavior [43, 44, 45]. The synthesis of M2‐PNA and the formation of the CPR were confirmed using matrix‐assisted laser desorption/ionization (MALDI) and gel electrophoresis, respectively (Figure 1a,b, Figures S1, S2 and Tables S1, S2). Multiple shifted bands were observed in the gel electrophoresis analysis, likely reflecting heterogeneous assembly states arising from membrane peptide oligomerization and multivalent PNA‐siRNA interactions. Similar higher‐order molecular forms of membrane‐associated M2 oligomers have previously been reported in SDS‐PAGE analyses of influenza M2 channels [46, 47]. As the M2TM‐PNA molar ratio increased, the intensity of the free siRNA band progressively decreased, indicating enhanced complex formation. Based on efficient siRNA complexation and reproducible liposome incorporation, a 3:1 M2TM‐PNA molar ratio was selected for the final CPR@LS formulation.

Subsequently, the CPR complex was embedded into the liposomes (LS) to form CPR@LS using a detergent‐mediated reconstitution process (Figure 1c) [48]. We calculated the theoretical maximum capacity of CPR on the surface of a liposome of a given size (Figure S3), and decided to use 20 CPRs per liposome as the proper number for further structural characterization of CPR@LS. The incorporation was confirmed by dynamic light scattering (DLS), which showed an increase in the hydrodynamic diameter from 75.4 ± 1.8 nm for bare liposomes to 99.3 ± 6.23 nm for CPR@LS (Figure 1d,e). Considering that nucleic acid duplexes exhibit an axial rise of approximately 3.4 Å per base pair, the contour length of the 31‐mer PNA‐siRNA duplex is estimated to be ∼10.5 nm. Assuming radial presentation of the duplexes on the liposome surface, the particle diameter would be expected to increase by approximately 21 nm relative to the bare liposome (Bare LS). This theoretical estimate agrees well with the experimentally observed DLS increase (∼24 nm), supporting the conclusion that the enlarged hydrodynamic diameter originates from surface‐displayed CPR complexes rather than nonspecific aggregation.

Additionally, circular dichroism (CD) spectroscopy demonstrated the helical structure of the M2TM component, as evidenced by distinct changes in the spectral profile. The increase in the A222/A208 ratio from 0.86 (in the dissolved state) to 1.48 (when embedded in the lipid bilayer of the liposome) suggests successful embedding of CPR, accompanied by the transformation of the M2TM structure into a tetramer within the lipid bilayer (Figure 1f) [29, 32]. Notably, this increased helicity remained consistent even when PNA and siRNA were individually conjugated to M2TM, respectively, with the spectrum of the siRNA duplex included within the helix spectrum of CPR@LS (Figure 1g). Collectively, these results confirm the complexation of the channel protein with siRNA via PNA and the effective embedding of the CPR complex into the liposome.

### 2.2 Cryo‐Electron Tomography Analysis of CPR@LS

**FIGURE 2:**
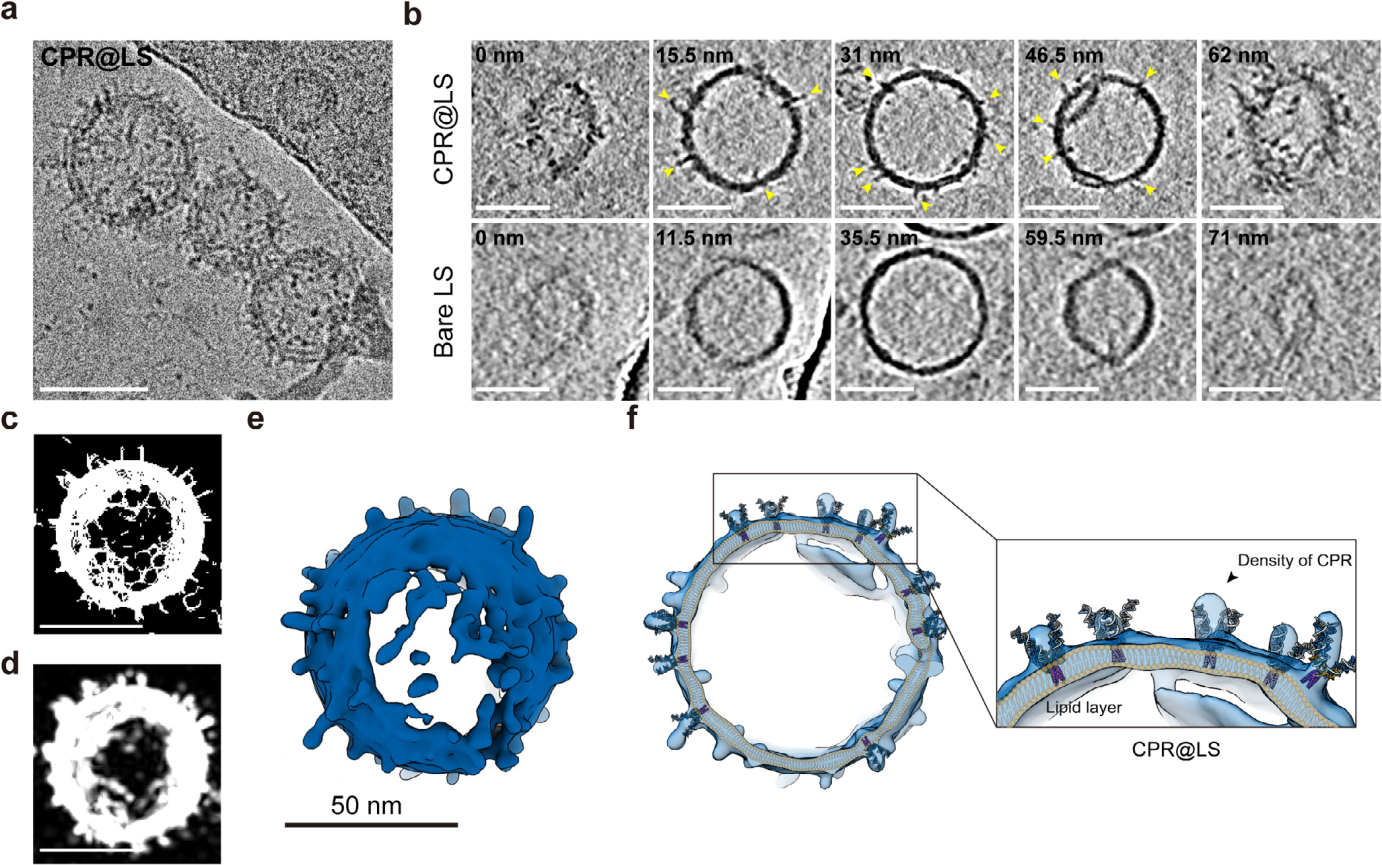
Cryo‐electron tomography analysis of assembled CPR@LS structures. (a) Representative cryo‐ET micrograph of CPR@LS. (b) Montage of tomographic slices through the reconstructed CPR@LS volume. Surface‐associated CPR densities are indicated by yellow arrowheads. Corresponding slices of Bare LS are shown for comparison. (c) Z‐projected image generated from representative tomographic slices. (d) Line‐recognition processed image highlighting surface‐associated CPR densities. (e) 3D tomographic reconstruction of CPR@LS. (f) Cross‐sectional view of the proposed CPR@LS structural model overlaid with the cryo‐ET density map, showing surface protrusions corresponding to CPR complexes. Scale bars represent 50 nm.

To confirm the structural integrity and surface organization of CPR@LS, we employed cryo‐electron tomography (cryo‐ET). The micrograph revealed distinctive density compared to the Bare LS (Figure 2a and Figure S4a). Tomographic slices of CPR@LS displayed distinct surface densities along the liposomal surface, indicated by yellow arrowheads, which were absent in Bare LS (Figure 2b and Videos S1, S2). These densities were observed across different z‐slice levels, each spaced 15.5 nm apart, demonstrating the surface localization of CPR complexes.

To further visualize the distribution of CPR‐associated densities, representative tomographic slices were subjected to Z‐projection and line‐recognition image processing (Figure 2c,d). The processed images highlighted multiple surface‐associated densities distributed around the liposome surface, with approximately 25 distinct surface protrusions observed on a representative liposome. This number is consistent with the intended formulation density of 20 CPR complexes per liposome, supporting the successful incorporation of CPR complexes onto the liposome surface.

To obtain a 3D reconstruction of CPR@LS, we recorded a tilt series over a ± 60° range in 3° increments, selecting 41 images to generate the montage. The tomographic reconstruction demonstrated that the CPR complexes on the liposome surface are well‐ distributed, visible as protrusions, unlike the clear surface morphology from the Bare LS (Figure 2e and Figure S4b,c). This reconstruction allowed us to propose a cross‐sectional view of the CPR@LS structural model, wherein the cryo‐ET density map exhibits protrusions corresponding in size and volume to individual CPR complexes (Figure 2f). Together, these observations support that CPR complexes are incorporated on the surface of the liposome.

### 2.3 Enhanced Endosomal Escape of CPR@LS Following Intracellular Uptake

To evaluate the endosomal escape efficiency of CPR@LS by the disruption of the endosomal pH gradient, an endosomal trafficking assay was conducted. Since the PNA and siRNA are conjugated on the N‐terminus of M2TM, the CPR complex is oriented unidirectionally, and the protons would be absorbed into the empty cavity of the liposome.

We first investigated whether the CPR complex on the surface of liposomes could enhance cellular delivery. It is known that the multivalent tethering of polyanionic ligands on the surface facilitated endocytosis into cells by interactions with class A scavenger receptors (SR‐As) [46].

**FIGURE 3:**
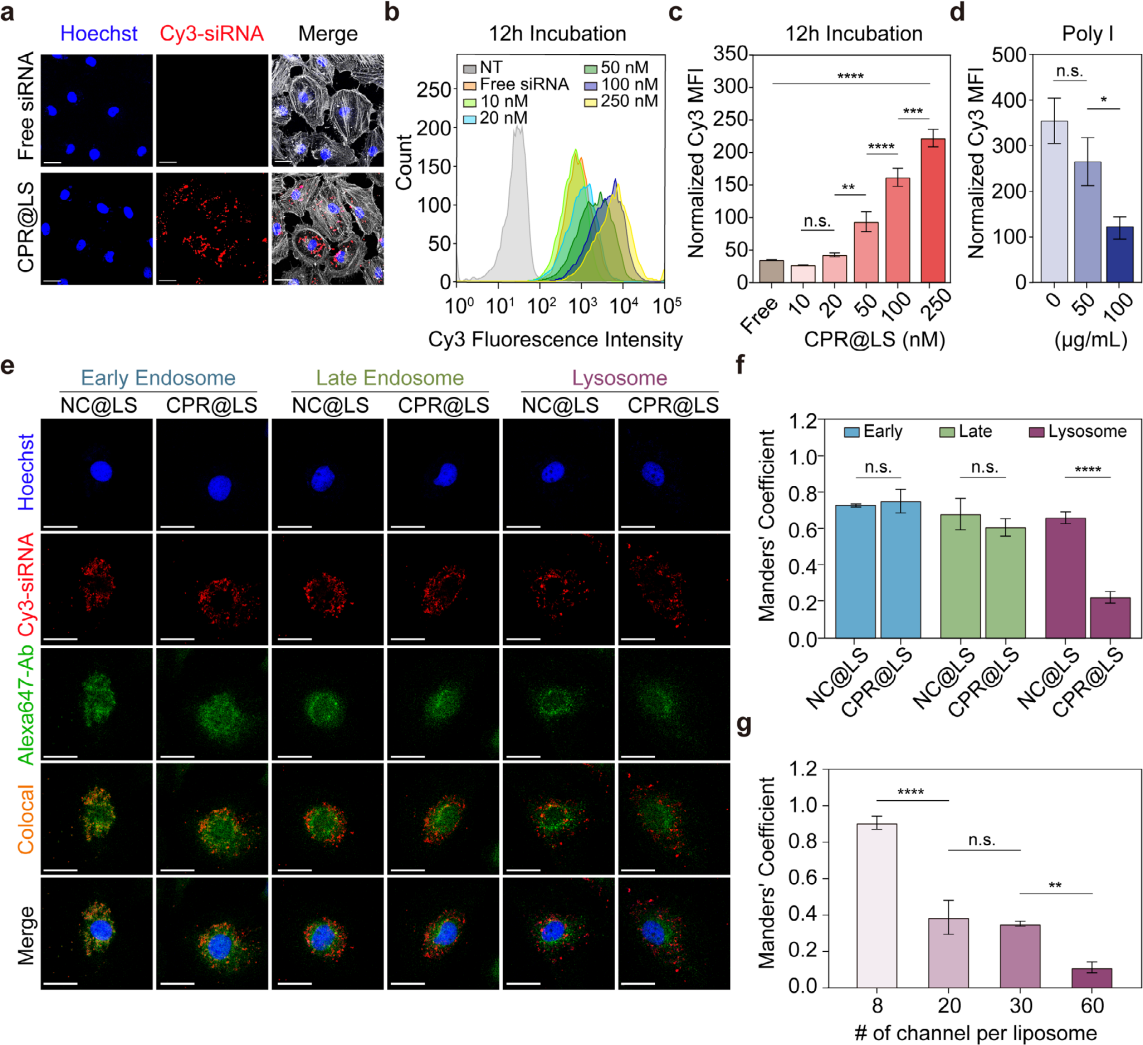
Enhanced endosomal escape efficiency following class A scavenger receptor (SR‐A)‐mediated endocytosis. (a) Confocal microscopy images showing enhanced intracellular uptake of CPR@LS compared to free siRNA after 12 h incubation. (b) Flow cytometry histograms demonstrating concentration‐dependent cellular uptake of CPR@LS after 12 h incubation. (c) Mean fluorescence intensity (MFI) quantification of the flow cytometry data shown in (b). (d) Effect of polyinosinic acid (Poly I), an SR‐A ligand, on the intracellular uptake of CPR@LS, quantified by MFI analysis. (e) Confocal microscopy images showing colocalization of Cy3‐siRNA‐containing CPR@LS or NC@LS with early endosomes, late endosomes, and lysosomes (Alexa647). (f) Manders’ coefficient quantification of colocalization shown in (e), analyzed using the JACoP plugin in ImageJ software. (g) Manders’ coefficient values of CPR@LS formulated with different numbers of M2TM channels per liposome. Statistical analysis was performed using one‐way ANOVA. ^*^ *P* ≤ 0.0332, ^**^ *P* ≤ 0.0031, ^***^ *P* ≤ 0.0002, ^****^ *P* ≤ 0.0001; n.s., not significant. Scale bars represent 30 µm.

In addition, the resulting surface architecture imposes steric and electrostatic hindrance, thereby enhancing resistance to nuclease‐mediated degradation [47, 48, 49]. This approach would be beneficial to overcome the inherently low cell penetration ability of siRNA drugs. To demonstrate SR‐A‐mediated intracellular delivery, we compared the cellular uptake of CPR@LS with that of free siRNA and further validated the mechanism by inhibiting uptake using SR‐A ligands. While maintaining constant thermal stability and minimal cytotoxicity to cells (Figure S5), confocal microscopy revealed substantially enhanced intracellular accumulation of CPR@LS compared to free siRNA after 12 h incubation at a concentration of 250 nM (Figure 3a). Flow cytometry histograms demonstrated a dose‐dependent increase in cellular uptake of CPR@LS after 12 h incubation (Figure 3b), and the corresponding MFI quantification showed a 3.5‐fold increase in intracellular uptake efficiency compared to free siRNA at 250 nM (Figure 3c). Similar concentration‐dependent uptake behavior was observed in confocal microscopy images acquired at 12 h (Figure S6a) and was further confirmed by a flow cytometry histogram at the 1 h time point (Figure S6b). To further elucidate the uptake mechanism of CPR@LS through interaction with SR‐As, we employed polyinosinic acid (Poly I) and fucoidan (FCD), which are binding ligands of SR‐As. Treatment with these pharmacological inhibitors resulted in a decrease in intracellular uptake of CPR@LS as the concentration of SR‐A ligands increased for cells with similar expression levels of SR‐As (Figure 3d and Figure S6c–e). To further validate the involvement of SR‐A in CPR@LS internalization, we compared cellular uptake in cell lines exhibiting different levels of SR‐A expression. Flow cytometric analysis confirmed substantially higher SR‐A expression in HeLa cells compared with HEK293 cells (Figure S7a). Consistent with this expression profile, CPR@LS uptake was significantly greater in HeLa cells than in HEK293 cells at both 10 and 100 nM concentrations (Figure S7b–d). Moreover, the extent of cellular uptake correlated with SR‐A expression levels, providing additional evidence that CPR@LS internalization is predominantly mediated through SR‐A‐dependent interactions rather than nonspecific membrane perturbation. These findings suggest that tethered siRNAs around the surface of liposomes play a crucial role as mediators for the cellular internalization of CPR@LS through SR‐As.

We then evaluated the endosomal escape efficiency of CPR@LS by an endosomal trafficking assay. This involved investigating the colocalization of CPR@LS with various endosomal compartments, including early endosomes, late endosomes, and lysosomes. Colocalization was assessed using confocal imaging with recombinant antibodies targeting endo/lysosomal compartment membrane proteins such as EEA1 for early endosomes, RAB7 for late endosomes, and LAMP1 for lysosomes. For the observation of proton conductivity through the channel, quantitative analysis based on Manders’ coefficient values was performed by comparing with NC@LS, a liposome embedded with a CPR containing the M2TM G34F mutation, which abolishes its ability to conduct protons [50]. Quantitative analysis based on Manders' coefficient values revealed similar colocalization among CPR@LS and NC@LS at early and late endosomes. However, CPR@LS exhibited approximately 40% lower coefficient values at lysosomes compared to NC@LS, indicating that CPR@LS induces endosomal escape during the endocytic process (Figure 3e,f). This trend was further supported by utilizing baculovirus expressing RAB5, RAB7, and LAMP1, where approximately 40% higher endosomal escape efficiency was observed compared to NC@LS (Figure S8). To further investigate the influence of proton channel number on endosomal escape, we varied the number of M2TM channels incorporated per liposome and quantified lysosomal colocalization using Manders' coefficient. Increasing the number of channels from 8 to 60 per liposome progressively decreased the lysosomal colocalization, indicating enhanced endosomal escape efficiency. Notably, liposomes containing 60 channels per particle exhibited approximately 9 fold lower lysosomal colocalization than those containing 8 channels, demonstrating a strong channel density‐dependent enhancement of endosomal escape (Figure 3g). We also evaluated the endosomal escape efficiency of the positive control, PC@LS. PC@LS exhibited less colocalization with recombinant LAMP1 antibody compared to CPR@LS (Figure S9).

### 2.4 Dual Target Gene Knockdown of CPR@LS

**FIGURE 4:**
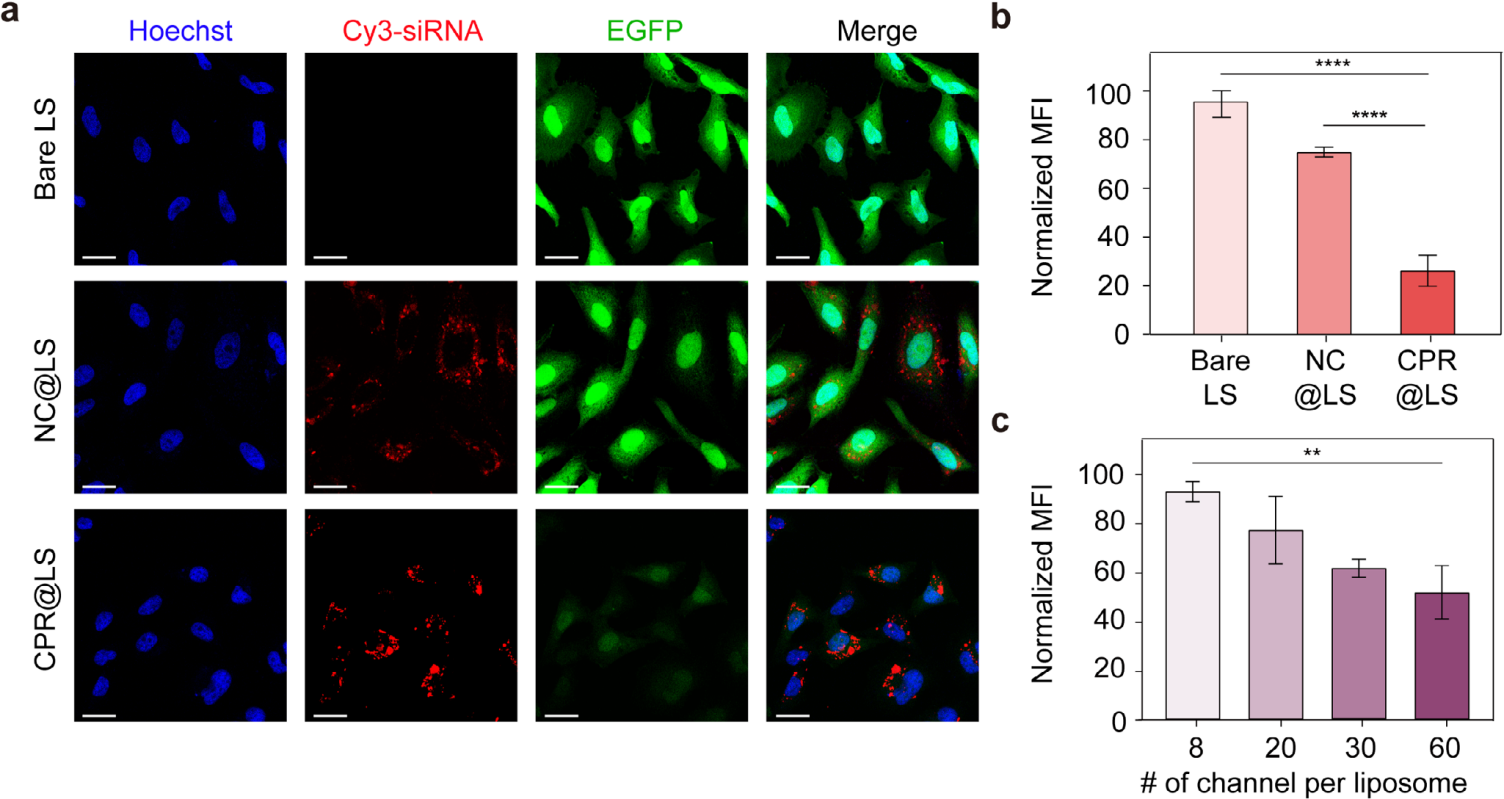
Effective gene knockdown induced by the M2TM channel. (a) Confocal images of HeLa‐EGFP cells demonstrating uptake and target EGFP gene knockdown by NC@LS and CPR@LS. (b) Normalized mean fluorescence intensity (MFI) of EGFP expressed from HeLa‐EGFP cells treated with Bare LS, NC@LS, and CPR@LS. (c) Comparison of EGFP knockdown efficiency among four CPR@LS constructions embedded with different numbers of channels. Statistical analysis was conducted using one‐way ANOVA. ^**^ *P*≤0.0031, ^****^ *P*≤0.0001. The scale bar represents 30 µm.

Following the endosomal escape of CPR@LS mediated by proton conductance through the M2TM channel, we investigated the gene silencing efficiency of tethered siRNAs. The silencing activity of CPR@LS was observed to be three times higher than that of NC@LS (Figure 4a,b) when tethered siRNAs targeting enhanced green fluorescent protein (EGFP) were administered to HeLa‐EGFP cells. The influence of proton conductivity on gene silencing was further demonstrated by a significant reduction in EGFP expression levels correlated with an increased number of M2TM per CPR@LS.

To ensure uniform activity of the CPR complex in intracellular uptake across all liposomes, the number of CPR complexes was controlled to be eight on each liposome, and additional bare M2TM was embedded in liposomes at a higher number. Notably, a nearly 50% increase in knockdown efficiency was observed when the number of M2TM channels was elevated to 60 compared to 8 (Figure 4c). To leverage the enhanced cellular uptake and release functionality of CPR@LS for a dual targeting system, we encapsulated payload siRNA within the interior of the liposome. CPR@LS was designed to maintain a uniform density of the CPR complex, with tethered siRNA featuring a scrambled sequence to eliminate its influence on gene knockdown.

The observed decrease in EGFP expression levels upon treatment with CPR@LS encapsulating siRNA targeting EGFP correlated with an increase in the number of M2TM channels. This suggests that the disruption of the endosomal pH gradient by CPR@LS can enhance delivery efficiency of encapsulated siRNA substances as well (Figure S10).

**FIGURE 5:**
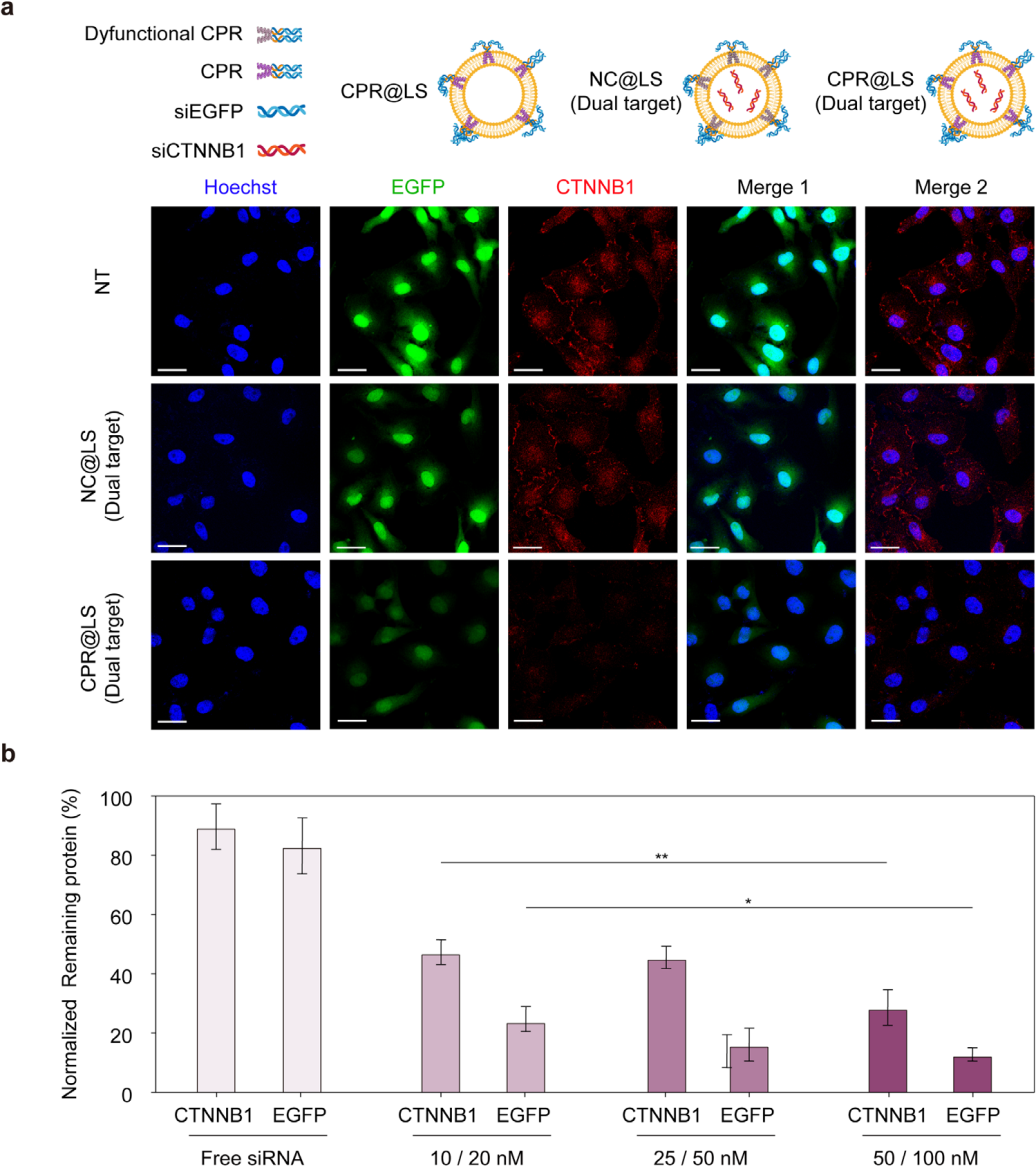
Dual gene knockdown of CPR@LS encapsulated with siRNA payload. (a) Confocal image showing simultaneous knockdown of dual genes, CTNNB1 and EGFP. (b) Western blot analysis of dual target gene knockdown by encapsulated siRNA targeting CTNNB1 and tethered siRNA targeting EGFP. Statistical analysis was conducted using one‐way ANOVA. ^*^ *P*≤0.0332, ^**^ *P*≤0.0031. The scale bar represents 30 µm.

To demonstrate the versatility of our dual targeting system, CPR@LS was designed to deliver tethered siRNA and encapsulated siRNA targeting the EGFP gene and the beta‐catenin (CTNNB1) gene, respectively. Confocal microscopy images acquired after a 6 h incubation of HeLa‐EGFP cells with dual‐targeting CPR@LS revealed a notable reduction in fluorescence intensity for both targeted genes, underscoring the potent dual gene delivery capability of CPR@LS (Figure 5a).

Additionally, EGFP and CTNNB1 band intensities simultaneously decreased, as analyzed by western blot, with each showing significant knockdown efficiency compared to the free siRNA with dose dependency (Figure 5b and Figure S11). Collectively, these findings emphasize the robust gene silencing ability of CPR@LS and highlight the importance of M2TM channel proton conductivity in enhancing the therapeutic effectiveness of siRNA drugs.

## 3 Conclusion

**FIGURE 6:**
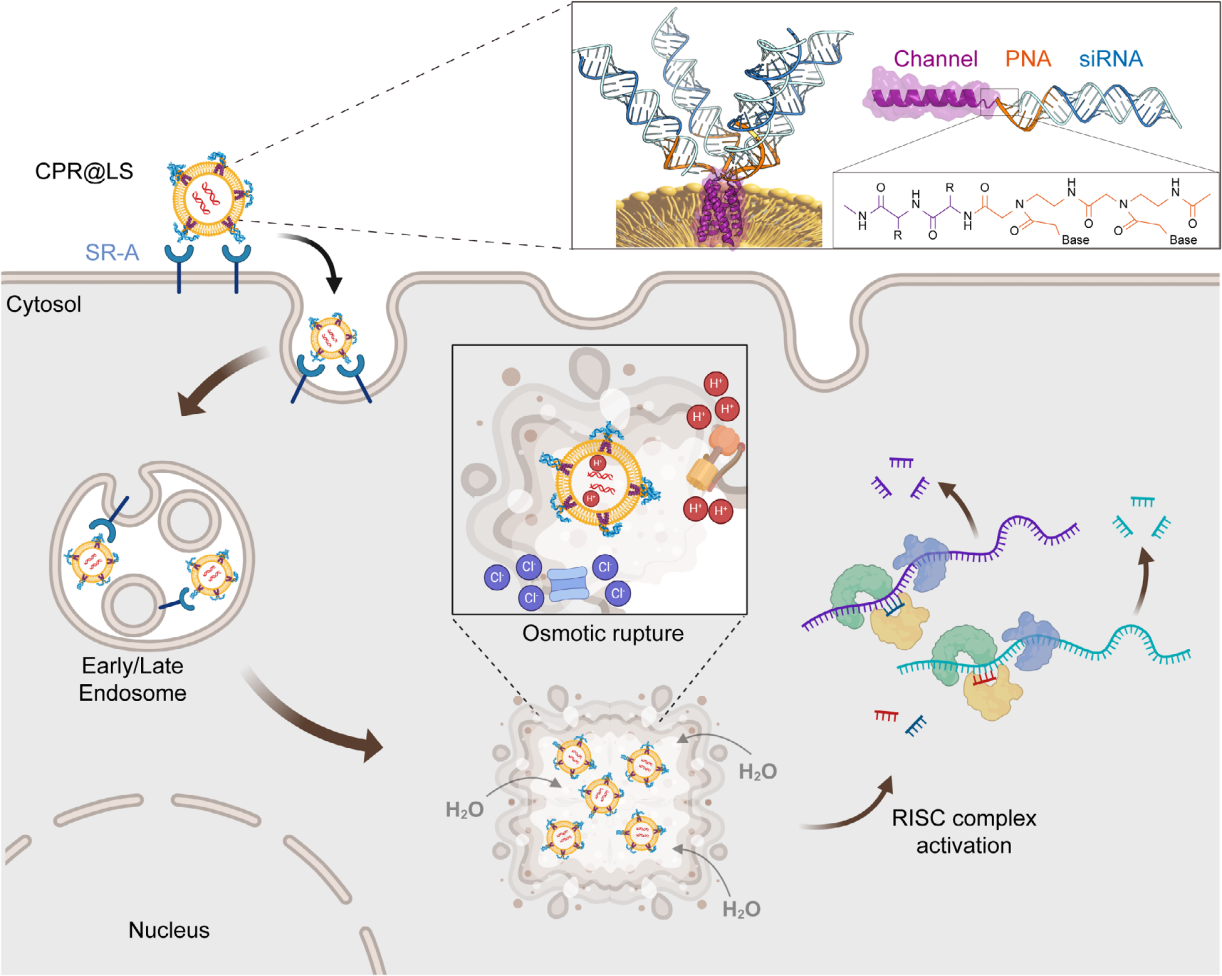
Proposed mechanism for enhancing siRNA delivery. Liposomes containing Channel‐PNA‐siRNA (CPR) complexes (CPR@LS) are endocytosed via the class A scavenger receptor (SR‐A). Inside the cells, the liposomes absorb protons through the CPR, leading to an increase in osmotic pressure by continuously conducting protons and chloride ions into the endosome. This process facilitates the release of both the encapsulated and surrounding siRNAs into the cytoplasm, thereby triggering RNA interference (RNAi) for gene silencing.

In this study, we addressed the critical barrier of lysosomal degradation in siRNA therapeutics by designing a proton channel‐PNA complex. By incorporating these complexes into liposomes (LS), termed CPR@LS, we achieved enhanced cellular uptake and facilitated endosomal escape. Unlike conventional carriers such as polyethylenimine (PEI) [37, 51] and polyamidoamine (PAMAM) [52] dendrimers, which rely on passive pH buffering via cationic polymers, our system mimics viral strategies by using a proton channel to actively drive proton influx into the liposomes. This unidirectional proton conductance triggers the osmotic rupture of endosomes containing CPR@LS (Figure 6).

Furthermore, we demonstrated multiplexed gene silencing by simultaneously delivering tethered and encapsulated siRNA payloads targeting distinct genes within a single liposomal platform. This dual‐delivery strategy highlights the versatility of CPR@LS as a modular siRNA nanocarrier capable of simultaneously regulating multiple intracellular targets. This approach offers a promising platform for minimizing intracellular degradation and broadening the applicability of advanced RNA therapeutics, including CRISPR/Cas‐based gene‐editing and gene‐suppression technologies [53, 54, 55]. Recent engineered extracellular vesicle and exosome‐based siRNA delivery systems have further demonstrated that improving intracellular trafficking, lysosomal escape, and tissue penetration substantially enhances therapeutic efficacy in complex disease environments [56, 57]. Collectively, these findings support CPR@LS as a bioinspired siRNA delivery platform capable of actively modulating intracellular trafficking and promoting efficient endosomal escape for enhanced cytosolic delivery. While our in vitro findings demonstrate significant mechanistic promise, successful in vivo translation will require navigating systemic physiological barriers. Future investigations must evaluate the platform's overall pharmacokinetic profile and the potential immunogenicity of the viral‐derived M2TM peptide during systemic circulation.

## 4 Experimental Section

### 4.1 Materials

The H‐Rink amid ChemMatrix resin (0.1 mmol, 0.45 mmol/g) was purchased from PCAS BioMatrix Inc. Dimethylformamide (DMF), methanol (MeOH), acetonitrile (ACN), isopropanol, and ethanol (EtOH) were purchased from Daejung. Amino acids and O‐(1H‐6‐chlorobenzotriazole‐1‐yl)‐1,1,3,3‐tetramethyluronium hexafluorophosphate (HCTU) were purchased from AAPPTec. Peptide nucleic acid (PNA) monomers were purchased from Panagene. Piperidine was purchased from Alfa Aesar. Diisopropylcarbodiimide (DIC), triisopropylsilane (TIS), trifluoroacetic acid (TFA), octyl β‐D‐glucopyranoside (OG), polyinosinic acid potassium salt (Poly I), fucoidan from Fucus vesiculosus, Triton X‐100, TWEEN 20, bovine serum albumin (BSA), and monoclonal anti‐β‐actin antibody were purchased from Sigma‐Aldrich. 30% bisacrylamide (29:1) solution, Tris‐HCl pH 8.8, Tris‐HCl pH 6.8, Bio‐Beads SM‐2 Resin, and 10% SDS solution were purchased from Bio‐Rad. 6x loading dye, high‐glucose Dulbecco's Modified Eagle Medium (DMEM), and penicillin‐streptomycin‐glutamine (100X) were purchased from Thermo Scientific. Synthesized siRNA was purchased from Bioneer. Recombinant Alexa Fluor 647 anti‐EEA1 antibody, recombinant Alexa Fluor 647 anti‐RAB7 antibody, recombinant Alexa Fluor 647 anti‐LAMP1 antibody, anti‐EGFP antibody, and anti‐CD204 antibody were purchased from Abcam. β‐Catenin antibody was purchased from Santa Cruz. 1 M Tris pH 8.0, 5 M NaCl, Alexa Fluor Phalloidin, CellLight Lysosomes (BacMam 2.0), CellLight Early Endosome‐GFP (BacMam 2.0), and CellLight Late Endosomes‐GFP (BacMam 2.0) were purchased from Invitrogen. 1‐palmitoyl‐2‐oleyl‐sn‐glycero‐phosphocholine (POPC), 1‐palmitoyl‐2‐oleyl‐sn‐glycero‐3‐phospho‐rac‐(1‐glycerol) (POPG), and an extruder were purchased from Avanti Polar Lipids. Sulfuric acid and PBS were purchased from Samchun. Dako Fluorescence Mounting Medium was purchased from Dako. A 4% paraformaldehyde (PFA) solution was purchased from Biosesang. A 0.5% trypsin‐EDTA (10X) and Opti‐MEM were purchased from Gibco. A 96‐well black plate was purchased from SPL Life Sciences.

### 4.2 Preparation of M2TM‐PNA

M2TM was synthesized using Fmoc‐solid phase peptide synthesis on H‐Rink amid ChemMatrix resin with a Syro I fully automated parallel peptide synthesizer (Biotage). The resin was swelled in DMF for 20 min, followed by repeated cycles of deprotection and coupling reactions. The standard peptide protocol was employed for deprotection, consisting of two steps: (1) treatment with 20% piperidine in DMF for 5 min, and (2) treatment with 40% piperidine for 10 min. For coupling, HCTU (5eq), NMM (10eq), and amino acid (5eq) in DMF were used for 8 min, and this process was repeated twice. After synthesis, the resin was washed with DCM and DMF five times each to remove unreacted reagents. PNA monomers were then sequentially synthesized at the N‐terminal of the peptide linked to the resin using the synthesizer. The standard PNA protocol was followed, with deprotection carried out in two steps: (1) treatment with 40% piperidine for 15 min, and (2) treatment with 20% piperidine for 15 min. Coupling was performed using HCTU (5eq), NMM (10eq), and amino acid (5eq) in DMF for 1 h and 30 min, and unreacted reagents were washed away using DCM and DMF.

The product was cleaved from the resin using a cleavage cocktail solution containing 95:2.5:2.5 of TFA:TIS:Deionized water in volume ratio for 2.5 h. The resin was removed by filtration, and the remaining solution was concentrated using nitrogen gas and precipitated with ice‐cold diethyl ether, then dried under vacuum to remove the ether. The dried pellet was purified using reverse‐phase high‐performance liquid chromatography (RP‐HPLC, Quaternary Gradient Module 2545, Waters) with a C4 reverse‐phase column and a linear gradient of 0.5%/ml, where the initial ratio of solvent A (distilled water/TFA, 99.9/0.1, vol%) to solvent B (isopropanol/ACN/distilled water/TFA, 60/30/10/0.1, vol%) was 6:4 in volume ratio. The molecular weight of M2TM‐PNA was confirmed by matrix‐assisted laser desorption /ionization‐time of flight (MALDI‐TOF) mass spectroscopy.

### 4.3 UV–Visible Spectroscopy

The molar concentration of M2TM‐PNA was analyzed by measuring the absorbance at 260 nm using an Agilent Technologies Cary 8454 UV–Visible spectroscopy. (Molar extinction coefficient of PNA sequence = 84300 L mol^−1^ cm^−1^, wherI260 nm (C) = 6600 L mol^−1^ cm^−1^, ε260 nm (G) = 11700 L mol^−1^ cm^−1^, ε260 nm (T) = 8800 L mol^−1^ cm^−1^, ε260 nm (A) = 13700 L mol^−1^ cm^−1^ acquired from PANAGENE Inc. PNA sequence: TCTTCTGCTT).

### 4.4 Formation of CPR Complex

M2TM‐PNA was dissolved in 1x annealing buffer (1x AB; 10 mM Tris, 20 mM NaCl at pH 8.0) containing 50 mM Octyl β‐D‐glucopyranoside (OG) detergent. The CPR complex was formed by annealing M2TM‐PNA with the siRNA duplex, which had been previously denatured on the heating block at 95°C for 2 min and slowly cooled down to room temperature at 50°C using the same process as the duplex. Considering the critical micelle concentration (CMC) value of OG, which is 20–25 mM, the final detergent concentration was 25 mM. The formation of the CPR complex was confirmed by electrophoresis on a 20% SDS polyacrylamide gel using the Gel‐Chemidoc XRS+ system.

### 4.5 Preparation of CPR@LS

POPC and POPG in chloroform were mixed in a 3:1 molar ratio, and the mixture was dried under a mild stream of nitrogen gas. 500 µl of 1x AB was added to the dried lipid film, and subsequently, a freeze‐thaw cycle was performed five times: (1) melt at 50°C, (2) freeze using liquid nitrogen, and (3) tip sonication for 1 min at 20% amplitude with 2 s on/off. The final lipid solution was then extruded 25 times through a 100 nm polycarbonate membrane filter, resulting in a final lipid concentration of 25 µmol/mL. The liposome was saturated by adding 400 µL of 22.8 mM OG in 1x AB to 200 µL of 13.4 mM lipid solution and incubating the mixture for 1.5 h at 4°C. Subsequently, 851.2 pmole of CPR complex dissolved in detergent was added to 95 µl of lipid solution and mixed with a Multi mixer SLRM‐3 (SeouLin Bioscience) in F3 mode at 10 rpm for 1.5 h at 4°C. After the incorporation process, the detergent was removed using Bio‐Beads. Before use, 0.5 g of dry beads were added to 20 mL of methanol and sonicated in a bath sonicator for 5 min to remove bubbles trapped in the beads. Methanol was decanted after spinning down the beads at 5000 rpm for 5 min, and this process was repeated using ethanol, deionized water, and 1x AB. By repeating this process five times, with each replacement of washed beads lasting 15 min, the final proteoliposome solution was centrifuged using a 100 kDa Amicon Ultra 0.5 mL filter at 15000 rpm for 5 min to remove unbound biomaterials, and finally, CPR@LS was prepared.

### 4.6 Colorimetric Assay

The remaining detergent was quantified by colorimetric assay. 25 µL of 20% 2,6‐dimethylphenol in absolute ethanol and 750 µl of sulfuric acid were added to 230 µL of sample with OG, and the mixture was incubated at room temperature for 40 min. Absorbance at 510 nm was measured by using UV–vis spectroscopy.

### 4.7 Dynamic Light Scattering

The hydrodynamic diameter (DH) of the nanoparticle was measured using a Malvern Zetasizer Nanoseries instrument. The size was calculated using the Malvern Zetasizer software, applying the Stokes‐Einstein equation. The polydispersity index (PDI) was calculated by dividing the standard deviation by the mean diameter. Samples for dynamic light scattering (DLS) were prepared at a concentration of 2.6 µM based on the lipid concentration in a buffer solution (10 mM Tris, 20 mM NaCl at pH 8.0) and were measured at 20°C.

### 4.8 Cryo‐Electron Tomography

Liposome‐containing Channel‐PNA‐siRNA (CPR) complexes (CPR@LS) were loaded onto a freshly glow‐discharged holey EM grid (Quantifoil R1.2/1.3 Cu 300 mesh). Sample vitrification was performed using a Vitrobot Mark IV (Thermo Fisher Scientific) at 4°C and 90% relative humidity. The tilt series was acquired using a Glacios cryo‐electron microscope (Thermo Fisher Scientific) operating at 200 kV and equipped with a Falcon 4i direct electron detector. Images were recorded at nominal magnification of x57 000, which corresponds to 1.83 Å/pixel at the specimen level, using Tomography software (v5.8, Thermo Fisher Scientific). At each batch tomography position, a tilt series with a tilt range of +/− 60° with 3° increments was recorded using a dose‐symmetric acquisition scheme. Defocus between −5 and −6 µm was applied. Approximately 2.5 e‐/Å2 electron dose was applied to each tilt image and recorded into 5 movie fractions. Using IMOD, poorly tracked tilt datasets were removed, and a total of 41 tilt series stacks were selected for tomogram reconstruction. The movie fractions were processed with AreTomo software for marker‐free tomographic alignment. During the alignment process, the tilt series were binned by a factor of 4, resulting in a pixel size of 7.32 Å. The tomogram reconstruction was performed using the simultaneous algebraic reconstruction technique (SART) in AreTomo [58]. The reconstructed tomograms were then processed with IsoNet software, which utilizes AI and deep learning techniques to denoise the data and reduce the missing wedge problem, resulting in denoised tomograms. These denoised tomograms were subsequently imported into EMAN2 (v2.91) for volume segmentation, from which 40 boxes containing recognizable features of the entire CPR@LS complexes were annotated and used to train neural network‐based tomogram segmentation [59].While training the neural network, other parameters were kept at default settings, but 200 training iterations and a batch size of 50 were applied. The segmented tomograms were filtered with a low‐pass filter to 30 Å and visualized using UCSF Chimera. The map contour level was arbitrarily adjusted so that densities corresponding to CPR@LS were separated from surrounding densities by visual inspection.

### 4.9 Circular Dichroism Spectroscopy

CD spectra of M2TM were acquired on a Jasco J 1500 spectropolarimeter. The CD spectra measurement was performed in quartz cells with 1 mm path length; the far UV light ranged from 200 to 240 nm.

### 4.10 Cell Culture

HeLa cells expressing the EGFP gene (HeLa‐EGFP cells) were sorted using an Aria Fusion Flow Cytometer for even expression of EGFP fluorescence intensity among cells. HeLa cells and HeLa‐EGFP cells were cultured in D10 (high glucose DMEM media containing 10% FBS, Penicillin 100 unit/ml, Streptomycin 100 µg/ml, and L‐glutamine 0.3 mg/ml) at 37°C under 5% CO_2_. The culture media was changed to fresh media at a cell confluency of 70–80%. After the removal of old media, cells were washed with 1x PBS. Then, 1.1x trypsin‐EDTA (TE) was added, and cells were incubated at 37°C under 5% CO_2_ for 3 min to detach the cells. Detached cells were spun down at 800 rpm for 5 min, and the pellet was resuspended in fresh media.

### 4.11 Cell Seeding

For the measurement of confocal microscopy, 200 µL of 4 × 10^4^ cells/mL HeLa cells with D10 media was added to an 8‐well chamber slide (Nunc Lab‐Tek II) and incubated overnight. For the flow cytometry analysis measured by BD FACS Melody Cell Sorter, 2 mL of 2 × 10^4^ cells/ml HeLa cells with D10 media was added to a 6‐well plate and incubated overnight. For the measurement with the Cytation 5 cell imaging multimode reader, 100 µL of 2 × 10^4^ cells/mL HeLa‐EGFP cells with D10 media was added to a 96‐well plate and incubated overnight. For the western blot assay, 1 mL of 1 × 10^5^ cells/mL HeLa‐EGFP cells with D10 media was added to a 12‐well plate and incubated overnight. For all the assays, D10 media was replaced with Opti‐MEM 2 h before the sample treatment.

### 4.12 Cellular Uptake Assay

CPR@LS was treated at 60% confluency, and HeLa cells were incubated for a specific time. After incubation, HeLa cells were fixed with 4% PFA. For the confocal image, nucleus and F‐actin were stained with Hoechst 33342 and Alexa Fluor Phalloidin.

### 4.13 Inhibition Assay for Mechanism Study of Cellular Uptake

50 and 100 µg/mL of polyinosinic acid (Poly I) and 500 and 1000 µg/mL of fucoidan (FCD) were treated to HeLa cells and incubated for 1 h. Then, CPR@LS was treated for a specific time at 37°C. Then, HeLa cells were fixed with 4% PFA and measured with confocal microscopy and flow cytometry.

### 4.14 Endosomal Trafficking Assay Using Recombinant Antibody

After treatment of CPR@LS, cells were incubated for 12 h at 37°C, cells were fixed with 4% PFA, and permeabilized with 0.05% Triton X‐100 for 1 min and blocked with 1% BSA in 0.05% Tween 20 for 1 h at room temperature. Then, endosomal compartments were stained with recombinant antibodyagainst EEA1, RAB7, or LAMP1 overnight at 4°C, and after washing, nuclei and phalloidin were stained.

### 4.15 Visualizing Endosomal Compartment using Baculovirus

A baculovirus containing plasmids for expressing RAB5, RAB7, and LAMP1 was used to visualize the endosomal compartment in HeLa cells. 30 min before treating the baculovirus, D10 media was replaced with Opti‐MEM. Virus particles at a concentration of 50 particles per cell were applied. Cells were then incubated overnight at 37°C, and after washing, CPR@LS was applied for 24 h at 37°C, followed by a final wash.

### 4.16 EGFP Knockdown Assay

CPR@LS and CPR@LS with a mutated sequence of M2TM as a dysfunctional channel were treated to HeLa‐EGFP cells with Opti‐MEM. After incubation for a certain time at 37°C, Opti‐MEM was replaced with D10 media for 48 h. Then, the media was removed, and cells were fixed with 4% PFA.

### 4.17 Encapsulation of siRNA Payload

Protamine sulfate and siRNA, each dissolved in 150 µL of PBS, were mixed. The mixture was incubated at 4°C for 30 min. Subsequently, the lipid film was resuspended with the mixture, followed by one freeze‐thaw cycle (1) freezing using liquid nitrogen, (2) melting at 50°C, and (3) tip sonication for 3 min at 20% amplitude with a 5‐s on/2‐s off cycle. The final lipid solution was then extruded 29 times through a 100 nm polycarbonate membrane filter.

### 4.18 Western Blot Assay

HeLa‐EGFP cells of each well in 12plate was lysed and protein concentration in the supernatant was observed by 10% SDS gel electrophoresis. Then, the gel was transferred to a PVDF membrane, and the membrane was incubated with primary and secondary antibodies. Finally, the band was observed using a Gel‐Chemidoc XRS^+^ system.

## Funding

This work was supported by the National Research Foundation of Korea(NRF) grant funded by the Korean government(MSIT) (No. RS‐2023‐NR077270). This research was supported by the Nano & Material Technology Development Program through the National Research Foundation of Korea(NRF) funded by the Ministry of Science and ICT(RS‐2024‐00444177). Use of cryo‐EM facilities of the NEXUS/CELINE consortium was supported by a National Research Foundation (NRF) of Korea grant RS‐2024‐00440289 /RS‐2024‐00440614.

## Conflicts of Interest

The authors declare no conflict of interest.

## Supporting information




**Supporting File 1**: smll74802‐sup‐0001‐SuppMat.docx.

**Supporting File 2**: smll74802‐sup‐0002‐VideoS1.mp4.

**Supporting File 3**: smll74802‐sup‐0003‐VideoS2.mp4.

## Data Availability

The data that support the findings of this study are available from the corresponding author upon reasonable request.
