# Supplementary material for: Active pH Modulation by Proton Channel‐Peptide Nucleic Acid Complex for Effective siRNA Endosomal Escape
Source: Small. 2026 Jul 23;22(53):e74802. doi: 10.1002/smll.74802 (PMC13592411; doi:10.1002/smll.74802)
Supplement: Supplementary file 1 [file SMLL-22-e74802-s001.docx]

Supporting Information

Active pH Modulation by Proton Channel-Peptide Nucleic Acid Complex for Effective siRNA Endosomal Escape

Seongmin Ga,^a,b,+^ Nam Hyeong Kim,^a,b,c,+^ Si Yeon Ryu,^a,b,+^ Kyeong Hyun Kim,^a,b^ Eun Sung Kang,^a,b^ Hayeon Bae, ^a,b^ Seungmin Ryu, ^e,f^ Suhyeon Kim,^a,b^ Yeontae Jang,^g^ Bok-Soo Lee,^a,b^ Ji Young Nam,^d^ Minah Suh, ^d , e, ,f^ Jaecheol Lee, ^d, g^ Seung-Gu Kang,^a,b,d,j^ Jaekyung Hyun,^g,h^ Huong T. Kratochvil,^i,*^ Yong Ho Kim,^a,b,d,e,j,*^

**Table S1**. Sequences and molecular weights of each peptide or peptide-PNAs. M2NC represents dysfunctional M2 and M2PC represents active M2.

| **Peptide** | **Sequence**  **(PNA: 5’ – 3’ / peptide: N – C)** | **Molecular weight** |
| --- | --- | --- |
| M2WT | SSDPLVVAASIIGILHLILWILDRL | 2728 |
| M2NC | SSDPLVVAASIIFILHLILWILDRL | 2818 |
| M2PC | SSDPLVVAASIIGILHLILWILTRL | 2714 |
| M2WT-PNA | TCTTCTGCTT SSDPLVVAASIIGILHLILWILDRL | 5370 |
| M2NC-PNA | TCTTCTGCTT SSDPLVVAASIIFILHLILWILDRL | 5460 |
| M2PC-PNA | TCTTCTGCTT SSDPLVVAASIIGILHLILWILTRL | 5356 |

**Table S2**. Sequences and molecular weights of siRNAs

| **siRNA** | **Sequence (5’ – 3’)** | **Molecular weight** |
| --- | --- | --- |
| SiEGFP_AS | UGAAGUUCACCUUGAUGCCGU | 6650.02 |
| siEGFP_SS | AAGCAGAAGAACGGCAUCAAGGUGAACUUCA | 10035.21 |
| siEGFP_SS_Cy3 | Cy3-AAGCAGAAGAACGGCAUCAAGGUGAACUUCA | 10667.21 |
| siScramble_SS | AAGCAGAAGAGACAACAACGUUACAUGGCUG | 10035.21 |
| siScramble_AS | CAGCCAUGUAACGUUGUUGUC | 6650.02 |
| siCTNNB1_SS_dTdT | CUGUUGGAUUGAUUCGAAAtt | 6672.09 |
| siCTNNB1_AS_dTdT | UUUCGAAUCAAUCCAACAGtt | 6598.08 |

**Figure S1.** Mass of synthesized M2TM and M2TM-PNA**.** Mass of (a) M2WT, (b) M2NC, (c) M2PC, (d) M2WT-PNA, (e) M2NC-PNA, and (f) M2PC-PNA were measured by MALDI-TOF mass spectrum.

**Figure S2.** Complexation of Channel-PNA-siRNA (CPR). Complexations with different ratios of siRNA and (a) M2NC-PNA, (b) M2PC-PNA were observed using 20% SDS gel.

**Figure S3.** Geometric model for estimating CPR loading capacity on liposomes. Theoretical calculation of CPR loading capacity on liposome surfaces based on effective steric footprint and hexagonal packing density.

**Figure S4**. Structural analysis of Bare LS using Cryo-ET. (a) Cryo-ET micrography of Bare LS showing homogenous particles without protruding densities. (b) Three-dimensional reconstructed montage of Bare LS based on tilt series stacks sorted by IMOD software. (c) Suggested model of Bare LS. The scale bar represents 50 nm.

**Figure S5.** Thermal stability and cytotoxicity of CPR@LS. Change in hydrodynamic diameters at 37 °C of (a) Bare LS and (b) CPR@LS for 3 days were measured by DLS. Cell viability after treatment of CPR@LS to HeLa cells, incubated at 37 °C for (c) 12 h and (d) 24 h was measured by Lactate Dehydrogenase (LDH) cytotoxicity assay.

**Figure S6.** SR-A-mediated intracellular uptake of CPR@LS. (a) Confocal microscopy images of HeLa cells treated with CPR@LS at different siRNA concentrations (10, 20, 50, and 100 nM) after 12 h incubation. (b) Flow cytometry histograms showing concentration-dependent cellular uptake of CPR@LS after 1 h incubation. (c) Effect of fucoidan (FCD), an SR-A ligand, on the intracellular uptake of CPR@LS, quantified by mean fluorescence intensity (MFI). (d) Confocal microscopy images of HeLa cells pretreated with Poly I or FCD prior to CPR@LS treatment. (e) Western blot analysis of SR-A expression levels in HeLa cells, HeLa cells treated with Poly I or FCD, and HeLa-EGFP cells. Statistical analysis was conducted using one-way ANOVA. *P≤0.0332, **P≤0.0031, ***P≤0.0002, ****P≤0.0001, n.s.: no significant difference. Scale bar represents 30 µm.

**Figure S7.** Validation of SR-A-dependent uptake of CPR@LS using cell lines with differential SR-A expression levels. a) Flow cytometric analysis of SR-A expression in HEK293 and HeLa cells using an anti-SR-A antibody. b,c) Representative flow cytometry histograms showing concentration-dependent uptake of CPR@LS in HEK293 and HeLa cells, respectively. d) Quantification of Cy3-labeled CPR@LS uptake in HEK293 and HeLa cells at the indicated concentrations. Statistical analysis was conducted using one-way ANOVA. *P≤0.0332, **P≤0.0031, ***P≤0.0002, ****P≤0.0001, n.s.: no significant difference. Scale bar represents 30 µm.

**Figure S8.** Endosomal escape tendency by visualizing endosomal compartment using baculovirus. (a) Confocal images showing colocalization between each endosomal compartment and NC@LS or CPR@LS. (b,c) Colocalization amount was quantified by Manders’ coefficient and Pearson’s coefficient analyzed using coloc_2 plugin in imageJ software. Statistical analysis was conducted using one-way ANOVA. *P≤0.0332, ***P≤0.0002, ****P≤0.0001, n.s.: no significant difference. Scale bar represents 30 µm.

**Figure S9.** Endosomal escape of non-active, wild-type, and active-CPR@LS in lysosomal compartment. Confocal microscopy images of HeLa cells treated with CPR@LS and immunostained with Alexa647-labeled antibodies against EEA1, RAB7, and LAMP1. Scale bar represents 30 µm.

**Figure S10.** Endosomal escape tendency different number of M2. Confocal images of HeLa cells treated with Cy3-labeled CPR@LS for 6 h, followed by staining with an Alexa647-labeled anti-LAMP1 antibody. Scale bar representing 30 µm. Confocal images of HeLa cells treated with Cy3-labeled CPR@LS for 6 h, followed by staining with an Alexa647-labeled anti-LAMP1 antibody.

**Figure S11.** Knockdown tendency of encapsulated siRNA depending on Channel number. (a) The number of CPR complex and M2TM among number variation. (b) EGFP expression levels of HeLa -EGFP cells treated with siEGFP, where the scrambled sequence was used for tethered siRNA.

**Figure S12.** Dual target gene knockdown. Western blot analysis of dual-targeting CPR@LS in a dose-dependent manner.
